# Up in the air: Threats to Afromontane biodiversity from climate change and habitat loss revealed by genetic monitoring of the Ethiopian Highlands bat

**DOI:** 10.1111/eva.13161

**Published:** 2020-12-07

**Authors:** Orly Razgour, Mohammed Kasso, Helena Santos, Javier Juste

**Affiliations:** ^1^ Biosciences University of Exeter Exeter UK; ^2^ School of Biological and Environmental Sciences University of Stirling Stirling UK; ^3^ Biology Department Dire Dawa University Dire Dawa Ethiopia; ^4^ Research Network in Biodiversity and Evolutionary Biology Research Centre in Biodiversity and Genetic Resources (InBIO‐CIBIO) Vairão Portugal; ^5^ Faculty of Sciences University of Porto Porto Portugal; ^6^ Estación Biológica de Doñana (CSIC) Sevilla Spain; ^7^ CIBER de Epidemiología y Salud Pública. CIBERESP Madrid Spain

**Keywords:** approximate Bayesian computation, bats, climate change, conservation genetics, endemic species, land‐use change, tropical montane forests

## Abstract

While climate change is recognized as a major future threat to biodiversity, most species are currently threatened by extensive human‐induced habitat loss, fragmentation and degradation. Tropical high‐altitude alpine and montane forest ecosystems and their biodiversity are particularly sensitive to temperature increases under climate change, but they are also subject to accelerated pressures from land conversion and degradation due to a growing human population. We studied the combined effects of anthropogenic land‐use change, past and future climate changes and mountain range isolation on the endemic Ethiopian Highlands long‐eared bat, *Plecotus balensis*, an understudied bat that is restricted to the remnant natural high‐altitude Afroalpine and Afromontane habitats. We integrated ecological niche modelling, landscape genetics and model‐based inference to assess the genetic, geographic and demographic impacts of past and recent environmental changes. We show that mountain range isolation and historic climates shaped population structure and patterns of genetic variation, but recent anthropogenic land‐use change and habitat degradation are associated with a severe population decline and loss of genetic diversity. Models predict that the suitable niche of this bat has been progressively shrinking since the last glaciation period. This study highlights threats to Afroalpine and Afromontane biodiversity, squeezed to higher altitudes under climate change while losing genetic diversity and suffering population declines due to anthropogenic land‐use change. We conclude that the conservation of tropical montane biodiversity requires a holistic approach, using genetic, ecological and geographic information to understand the effects of environmental changes across temporal scales and simultaneously addressing the impacts of multiple threats.

## 1 INTRODUCTION

Clusters of alpine high mountain ecosystems that are isolated by different environmental conditions in the intervening lowlands can behave as authentic archipelagos. These sky islands contain unique diversity and provide natural laboratories to study evolution, speciation and the effects of habitat fragmentation (Hedberg, 1969; McCormack et al., 2009). Exceptionally high levels of endemism are attributed to differentiation processes resulting from climatic fluctuations during the Quaternary (Williams et al., 2004). Colder conditions during glacial periods led to the expansion of high‐altitude alpine and montane forest ecosystems into lower elevations, thus facilitating connectivity between previously isolated populations (Hewitt, 2000). On the other hand, these ecosystems are especially sensitive to current and future climate change, and as a result of climatic warming their area is rapidly diminishing (Moritz & Agudo, 2013), a process which will increase the isolation and extinction risk of their associated flora and fauna. This process is expected to be particularly intense in the scarce alpine environments distributed at high elevations across the tropics. Tropical mountains are predicted to experience the highest levels of extinctions and changes to fauna assemblages under future climate change (Lawler et al., 2009). High‐altitude species living close to mountain tops may lose their entire range as their suitable climatic regime and its associated ecosystem disappears with increasing temperatures (Pimm, 2009; Williams et al., 2007).

**Figure 1:**
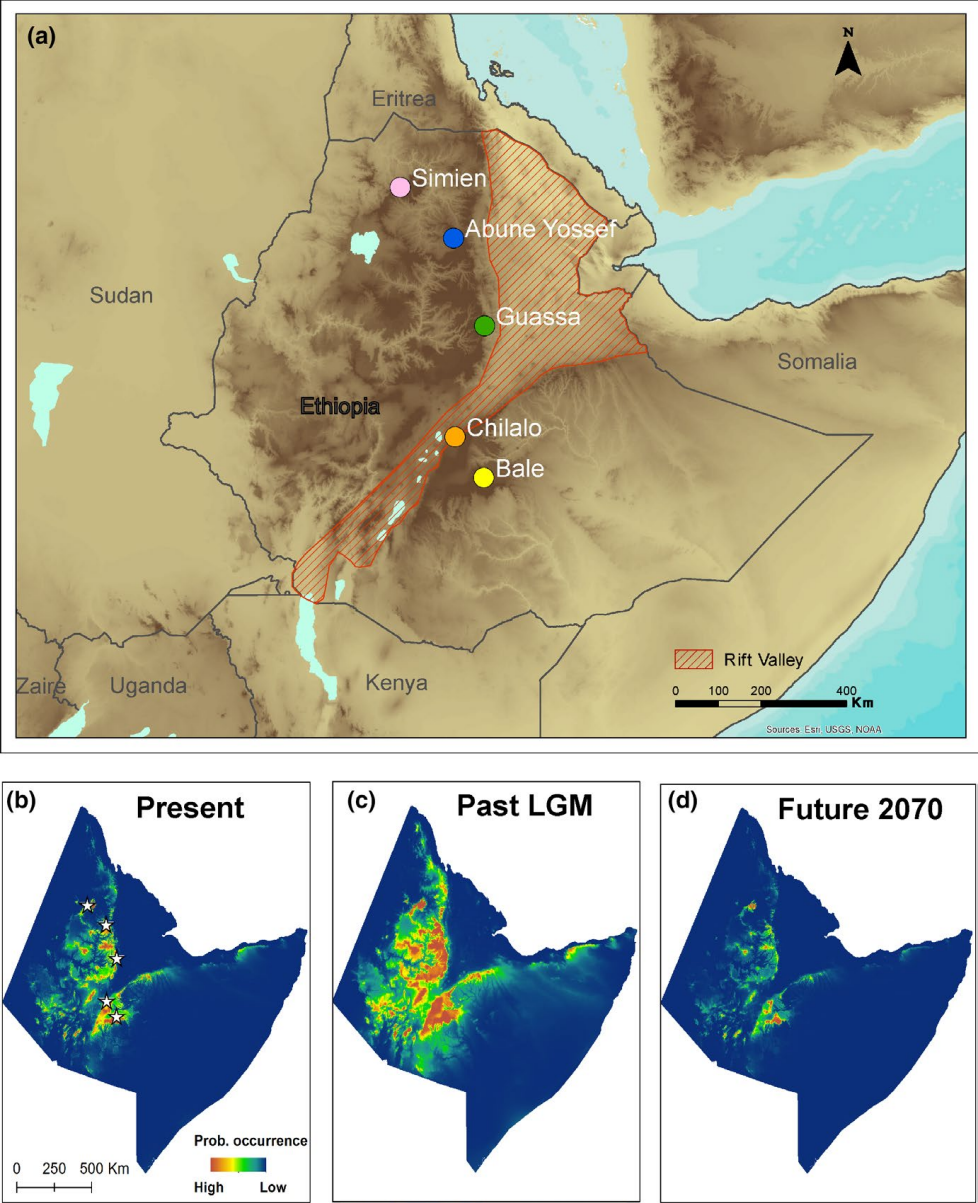
(a) Map of the five sampled mountain ranges in Ethiopia presented over a digital elevation model with the location of the Rift Valley marked with red stripes. b‐d) Projected probability of occurrence for *Plecotus balensis* across the horn of Africa based on ecological niche modelling outputs for (b) present, (c) past (Last Glacial Maximum ~ 21K ya), and (d) future (2070) severe emissions scenario (RCP 8.5) climatic conditions. Probability of occurrence ranges from high in orange to low in blue; stars denote the location of mountain ranges sampled in this study

This problem is especially acute in understudied and highly threatened areas like the Ethiopian Highlands, where accelerated land conversion and degradation is placing further pressures on biodiversity (Yalden et al., 1996). The Ethiopian Highlands are a vast extent of high ground rising up to 4,620 masl with a low‐altitude limit of 1,500 masl. They cover an area of 519,278 km^2^ in the otherwise arid zone of the Horn of Africa and include 73% of the Afroalpine biome (areas above 3,200 masl) in Sub‐Saharan Africa (Williams et al., 2004). The Great Rift Valley crosses the Ethiopian central highlands dividing them into two main blocks, north‐east and south‐west (Ebinger et al., 2000) (Figure 1).

The Afroalpine biome shows one of the highest rates of endemism in the world due to a combination of their relative small areas, high isolation and climatic history (Reyes‐Velasco et al., 2018; Williams et al., 2004). These endemics are either of Palearctic (e.g., the Ethiopian ibex, *Capra walie,* the Ethiopian long‐eared bat, *Plecotus balensis*) or Afrotropical origin (e.g., geladas monkeys, *Theropithecus gelada,* the mountain nyala, *Tragelaphus buxtoni*) (Harmsen et al., 1991). Rapid human population growth in Ethiopia over the past 150 years, from approximately 6.6 million in 1868 (Nyssen et al., 2014) to more than 100 million (UN, 2019), and the corresponding increase in pressure on natural environments and human encroachment into national parks and other protected areas has resulted in extensive habitat degradation (Kidane et al., 2012). This pressure has been implicated with biodiversity losses, from the decline of endemic frogs (Gower et al., 2013) to the decline of large flagship mammal species such as the endemic Ethiopian wolf, *Canis simensis* (Stephens et al., 2001). However, some signs of vegetation recovery have been observed since the early 21st century following the initiation of environmental recovery programs in the 1980s and the establishment of new protected areas closing up to 15% of the country to livestock grazing (Nyssen et al., 2014).

Recently, a new species of the long‐eared bat genus *Plecotus* was described in the Ethiopian Highlands above 3,000 masl, the Bale long‐eared bat, *Plecotus balensis*. This bat is endemic to Ethiopia and possibly Eritrea (Juste et al., 2004; Kruskop & Laverenchenko, 2000) and is known by only a few specimens from just seven localities (Benda et al., 2004). The genus *Plecotus* is widely distributed across the Palearctic, and is characterized by high cryptic diversification and specific altitudinal and climatic associations (Spitzenberger et al., 2006). Due to their limited long‐distance dispersal abilities and habitat specializations, *Plecotus* bats are particularly sensitive to habitat loss (Razgour et al., 2014) and the effects of climate change (Razgour et al., 2013).

We use this recently described high‐altitude bat to determine how sky islands and historic, current and future environmental changes shape the distribution, genetic diversity and conservation status of tropical montane biodiversity. We project ecological niche models (ENMs) across temporal scales to track the effect of past climate changes on range suitability for *P. balensis* and predict impacts of future changes. We integrate approximate Bayesian computation inference of demographic history with landscape genetics analysis and markers with different mutation rates to disentangle the impact of historic vicariance events versus recent anthropogenic habitat loss and degradation on population size, genetic diversity and genetic connectivity of sky island biodiversity. Our study highlights threats to tropical montane biodiversity due to the combined effects of multiple stressors, being squeezed into higher altitudes due to climate change while losing genetic diversity and suffering population declines due to anthropogenic land‐use change.

## 2 MATERIALS AND METHODS

### 2.1 Sampling design

Bats were sampled during 2014–2015 in five isolated mountain ranges across the Ethiopian Highlands: (a) Bale Mountains National Park, the largest and southernmost massif that covers partially the most extensive Afroalpine area in the continent (Hillman, 1986), (b) Arsi Mountains National Park of Chillalo‐Galama Mountains, (c) Guassa Community Conservation Area, (d) Mount Abune Yosef, and (e) the Simien Mountains National Park (Figure 1a). The Ethiopian Highlands include unique ecosystems typically characterized by Afromontane forests up to 3,200 masl, Afroalpine wet and dry grasslands above 3,500 m and Afroalpine moorland habitat above 4,000 m. The Afromontane forest habitat includes the endemic Ethiopian juniper (*Juniperus procera*) and African redwood (*Hagenia abyssinica*) in the drier northern slopes and Erica (*Erica arborea*) and yellow wood (*Podocarpus* sp.) forests in the warmer and more humid southern slopes. We obtained 3 mm wing biopsies from 50 *Plecotus balensis* bats from 19 sites (Table S1). Bats were caught in two distinct ecoregions: Afromontane woodland (2,795–3,492 m; 30 bats) and Afroalpine grasslands and moorlands (3,773–4,224 m; 20 bats). In Bale, a sufficient number of bats were caught in each ecoregion type to separate the mountain range into two populations, “Bale‐D” (Dinsho) for Afromontane woodland and “Bale‐S” (Sanetti Plateau) for Afroalpine moorlands. Because only three individuals were caught in Chillalo‐Galama Mountains, samples from this mountain range could only be included in individual‐based analyses (ecological niche models, phylogenetic tree, haplotype network and population structure), as well as the demographic history analysis as part of the southern population. Three additional samples collected from two sites around the Abune Yosef Mountain in a former expedition in 1996 were also included in individual‐based analyses only.

### 2.2 Ecological niche modelling

Ecological niche models (ENMs) were generated with the programme MaxEnt v3.4.1 (Phillips et al., 2006) to determine the potential geographic distribution and ecological requirements of *P. balensis* across the Horn of Africa. The Horn of Africa was selected as the study area because it represents a biotic region that was likely accessible for the species over the relevant time periods covered by the modelling. As only 14 unclustered location records are available for this understudied species, we used a bias layer to account for potential uneven sampling efforts and low sample sizes. We assigned a value of 10 for mountain ranges surveyed during our expeditions and a value of 1 to the remaining study area. These values were used by Maxent to give weights to random background data during the modelling process so that pseudo‐absences better reflect potential geographic bias in sampling efforts (Fourcade et al., 2014). Model resolution was set at ~1 km (30 arc sec). We generated two types of models. The full model included a combination of climatic, topographic, land cover, vegetation cover, ecoregions and human footprint variables (Appendix A). The climatic model included only climatic and static topographic variables that could be projected to the future (ruggedness and slope). We removed highly correlated variables [*R* > |0.75|; tested with ENMTools v1.3 (Warren et al., 2010)] and variables that did not contribute to model gain (Table S2 for model variables). Final models included a regularization value of 2 and three features (linear, quadratic and product), selected based on AICc scores obtained with ENMTools, and 1,500 iterations. Model testing was carried out with 100 bootstrap replications using 20% of data for model testing. Output maps were converted to binary maps based on the thresholding method that maximizes sensitivity and specificity (Liu et al., 2013). Areas with values that fell above the threshold were considered as suitable for the bat.

Models were hind‐casted to the Last Glacial Maximum (LGM) and mid‐Holocene, and projected to the future (2070) using three General Circulation Models (CCSM4, MIROC‐ESM, MPI‐ESM‐LR) for each time period, and two Representative Concentration Pathways (RCP) scenarios for future projections only, the “worst case” scenario, RCP + 8.5 W/m^2^, and the more moderate RCP + 4.5 W/m^2^ scenario (IPCC, 2013). In our projected models we included the only climatic variable that affected the distribution of *P. balensis*, maximum temperatures (BIO5, WorldClim; see “Ecological niche models” section in the Results) and a static topographic variable, topographic ruggedness (calculated from SRTM map by computing the maximum elevation difference within a 5 km buffer around each cell using range statistics in ArcGIS v10.3.1, ESRI).

### 2.3 Generating the genetic datasets

Genomic DNA was extracted from all wing biopsy samples. The extraction protocol consisted of DNA precipitation with isopropanol after purification using saline precipitation. We selected three markers with different mutation rates, covering events that occurred during the Pleistocene (mitochondrial DNA fragments) versus recent‐contemporary impacts from the last few decades‐centuries (microsatellites). Two mitochondrial DNA (mtDNA) fragments were amplified as follows: a 650 bp fragment of the gene cytochrome b (cytb) using the primers MOLCIT‐F (Ibáñez et al., 2006) and MVZ16 (Smith & Patton, 1993); and a 460 bp fragment of the hyper‐variable region (HV1) of the control region using the primers L15926 and CSBF‐R (Wilkinson & Chapman, 1991). Samples were also genotyped for 19 polymorphic autosomal microsatellite loci previously developed for the genus (Razgour et al., 2013) (Appendix A for PCR reactions and sequencing). All microsatellite loci retained in the analysis did not depart from Hardy‐Weinberg equilibrium expectations, were not in linkage disequilibrium and had a low frequency of null alleles in most populations [tested with GENEPOP v4.2 (Rousset, 2008) and CERVUS v3.0.3 (Kalinowski et al., 2007)]. For power analysis for the genetic dataset see Appendix A.

### 2.4 Genetic data analysis

Mitochondrial DNA sequences from the cytb and HVI regions were aligned and concatenated into a single sequence (1,110 bp) with BioEdit v7.2.0 (Hall, 1999) and collapsed into haplotypes with DAMBE v6 (Xia & Xie, 2001). Bayesian phylogenetic trees were constructed for the concatenated cytb and HVI sequences in MrBayes v3.2.1 (Ronquist et al., 2012), using *Plecotus austriacus* as outgroup to root the tree. We ran 4x10^7^ generations with four chains, sampled every 200th generation, and two simultaneous runs, discarding the first 25% of trees as burn‐in. We used the Hasegawa–Kishino–Yano (HKY) model of DNA substitution with proportion of invariable sites model of rate variation for the cytb sequences and the HKY model with gamma‐distributed rate variation for the HVI sequences [selected by jModelTest2 v0.1.10 (Darriba et al., 2012) based on BIC values]. Trees and posterior probabilities were visualized with Figtree v1.3.1 (http://tree.bio.ed.ac.uk/software/figtree/). We calculated nucleotide polymorphism, haplotype diversity, genetic divergence and differentiation between populations in DnaSP v5.10 (Librado & Rozas, 2009).

Population genetics summary statistics for the microsatellite dataset were performed in GenAlEx v6.4 (Peakall & Smouse, 2006) and FSTAT v2.9.3.2 (Goudet, 1995) correcting for differences in sample sizes. Levels of inbreeding within each population were calculated using the TrioML measure in Coancestry (Wang, 2011). Linear models were performed in R (CRAN) to related levels of genetic diversity (allelic richness) and inbreeding to NDVI during the dry season, human footprint index and cover of key land‐use types likely to either positively (forest) or negatively (arable land) affect habitat quality for bats. Cover of the land‐use types was measured in ArcGIS v10.3.1 (ESRI) within 5 km buffers around population capture locations using the GlobCover 2009 map. Values of NDVI and human footprint index were averaged across the 5 km buffers. We used 5 km buffer around capture sites to reflect the potential home range of the species, estimated based on the home range size of its better‐studied cryptic congener *Plecotus austriacus* (Razgour et al., 2011).

Extent of genetic differentiation between populations was calculated using two measures: F_ST_ in SPAGeDi (Hardy & Vekemans, 2002) and Jost's D (Jost, 2008) in GenAlEx v6.4. Individual‐based Bayesian assignment tests implemented in STRUCTURE v2.3.3 (Pritchard et al., 2000) were used to infer genetic population structure, varying number of clusters (K) from 1 to 10, with 10 replicates and 10^6^ Markov Chain Monte Carlo (MCMC) generations following a burn‐in phase of 5 × 10^5^ generations. The number of distinct clusters was determined using STRUCTURE HARVESTER (Earl & vonHoldt, 2012) based on Evanno's delta K method and mean log‐likelihood. Cluster assignment was visualized with DISTRUCT (Rosenberg, 2003).

### 2.5 Landscape genetics analysis

We used the landscape genetics approach to determine how landscape heterogeneity affects functional connectivity and gene flow between mountain range populations of *P. balensis*. The analysis included landscape variables that were deemed to impede or facilitate movement in this bat given what is known about its habitat use and geographic distribution, including topography, ecoregions, tree cover, land cover, streams, human footprint and night lights. Landscape variables were converted to resistance surfaces in ArcGIS and were assigned different resistance costs based on knowledge of the ecology of this species and its better‐studied cryptic congeners. Specifically, based on capture records from this and previous studies we know *P. balensis* is associated with high altitudes, high tropical montane forests, moorlands and alpine grasslands and is not found in urban areas. Based on ENMs generated in this study (see Results), we infer that *P. balensis* is associated with high topographic ruggedness and montane ecoregions. Finally, based on its wing morphology and cryptic congeners, this species is likely to be highly manoeuvrable, slow flier that relies on vegetation cover for protection from predators when commuting (Entwistle et al., 1996). Resistance costs ranged from one, no resistance to movement, to 100, strong barrier to movement (Table S3 for list of variables, their sources and different allocated resistance costs). Circuitscape v4.0.5 (McRae, 2006) was used to calculate resistance distance matrices between populations based on the cumulative cost of movement due to landscape resistance. We used multiple regression on distance matrices in the R package ecodist (Goslee & Urban, 2007) to select the best combination of resistance costs for each landscape variable based on strength of correlation with genetic distance (F_ST_ and Jost's D) between populations (Table S4).

We compared nine candidate sets of hypotheses for the effect of the landscape on genetic connectivity: topographic (altitude), land‐use (land cover map), anthropogenic (human footprint index), ecoregions, forest (per cent tree cover), hydro (distance to streams), hydro‐land (streams + land cover), anthropo‐eco (human index + ecoregions) and anthropo‐topo (human index + altitude). We used the Maximum Likelihood Population Effect (MLPE) approach (Van Strien et al., 2012) and the R packages lme4 (Bates et al., 2015) and usdm (Naimi et al., 2014). To reduce model collinearity, we only included variables with VIF < 4 in each hypothesis tested. We used AICc and BIC evidence weights to identify the best‐supported models. To account for the effect of geographic distance on genetic connectivity (MRDM for Euclidian distance and F_ST_: *R* ^2^ = 0.657, *p* = .035), we divided the log measure of genetic distance (F_ST_ or Jost's D) by log Euclidean distance between populations. All variables were log transformed to comply with model assumptions of normal distribution.

### 2.6 Approximate Bayesian computation (ABC) inference of evolutionary history

The demographic history of *P. balensis* was reconstructed using the ABC approach implemented in DIYABC v2.1.0 (Cornuet et al., 2014) in order to identify whether recent changes in population size have occurred in response to anthropogenic land‐use change and habitat fragmentation and degradation. Small sample sizes meant we could not compare the evolutionary history of each mountain range, but only the wider geographical areas. Therefore, we divided the dataset into samples from the north‐west (*n* = 28) versus south‐east (*n* = 22) of the Rift Valley due to its role in population structure (see Results section). We tested four competing scenarios of population changes: (a) a large ancient population split into two smaller populations, no change in population sizes; (b) population expansion after split; (c) after population split, both populations declined recently; and (d) after population split recent decline in the south‐eastern population only (Figure S1 for scenarios). Population split dates were kept flexible, ranging from pre‐post LGM (200–200,000 years ago), while recent decline dates were set at 20–1,000 years ago.

ABC analysis was carried out on the combined mtDNA and microsatellite datasets, including only recent samples (from 2014–2015). MtDNA substitution model and parameters were defined based on jModelTest2 results. We simulated 10^6^ datasets per scenario tested, and included most available summary statistics (18 in total). The posterior probability of scenarios was estimated using a weighted polychotomous logistic regression. We empirically evaluated the power of the model to discriminate among scenarios (confidence in scenario choice) by simulating pseudo‐observed datasets with the different scenarios and calculating false allocation rates. We evaluated model specificity (type 1 error) by simulating 500 pseudo‐observed datasets with the scenario selected by the ABC analysis and calculating rates of false scenario assignment. Model sensitivity (type 2 error) was calculated based on the proportion of 500 pseudo‐observed datasets simulated with other scenarios that were assigned to the scenario selected by the ABC analysis. We carried out model checking for the most probable scenario through performing a PCA on 1,000 simulated datasets generated from posterior parameter distributions and the observed dataset. We evaluated bias and precision on parameter estimation by calculating the relative mean bias and Relative Median Absolute Deviation (RMedAD) based on comparing 500 pseudo‐observed simulated datasets to 10,000 simulated datasets closest to observed dataset of each parameter (Cornuet et al., 2010, 2014).

## 3 RESULTS

Of the 50 bats captured during this study, 39 were males, five of which were sub‐adults, and only 11 were females, four of which were reproductive (lactating). All females and sub‐adults were caught exclusively in *Juniperus procera* or *Erica arborea* forests. Only adult males were caught at the higher altitude Afroalpine moorlands (Table S1).

### 3.1 Ecological niche models

Models had very high discrimination ability (AUCtrain = 0.997; AUCtest = 0.995 ± 0.01). The main variables affecting the probability of occurrence of *P. balensis* were maximum temperatures (highest contribution to model and highest effect on gain when removed), Ethiopian Afroalpine moorland and Ethiopian montane grassland and woodland ecoregions, high topographic ruggedness and forest land cover (Table S2). All other variables, including anthropogenic impact (human footprint), contributed very little to the model (<0.5%), and were therefore removed. The model projected high probability of *P. balensis* occurrence (i.e., falling above the threshold that maximizes model sensitivity and specificity) across the Ethiopian Highlands, split into mountain ranges to the north and south of the Rift Valley, as well as mountain ranges in Eritrea and Somaliland (Figure 1; Figure S2). However, suitable areas covered less than 1% of the Horn of Africa. Climatic models projected across temporal scales predicted that the climatically suitable range was 4.5 times larger during the LGM and 3.8 times larger during the mid‐Holocene. Only a quarter of the current range was projected to remain suitable by the end of the century, equating to 5.6% of the LGM suitable range (based on the more severe RCP 8.5 scenario; Figure S2; Table 1).

**Table 1:** Projected range changes for *Plecotus balensis* from the last glacial maximum (LGM) until the end of the century (2070)

Model	Suitable cells	% horn of Africa suitable	% range change
Present	18,036	0.76	
Future (2070; RCP 8.5)	4,623	0.19 (0.11–0.22)	−74.4 (71.3–85.5)
Future (2070; RCP 4.5)	6,832	0.29 (0.25–0.36)	−62.1 (52.7–67.4)
LGM	81,815	3.54 (2.82–4.01)	353.6 (263.6–410.1)
Mid‐Holocene	68,065	2.93 (1.86–3.69)	277.4 (142.4–372.3)

Note

Presented values are from the ensemble ENMs with range of values from models generated with the different GCMs in brackets. Per cent range change indicates the per cent by which the present suitable range has increased or decreased under projections for different time scales

### 3.2 Genetic composition

**Figure 2:**
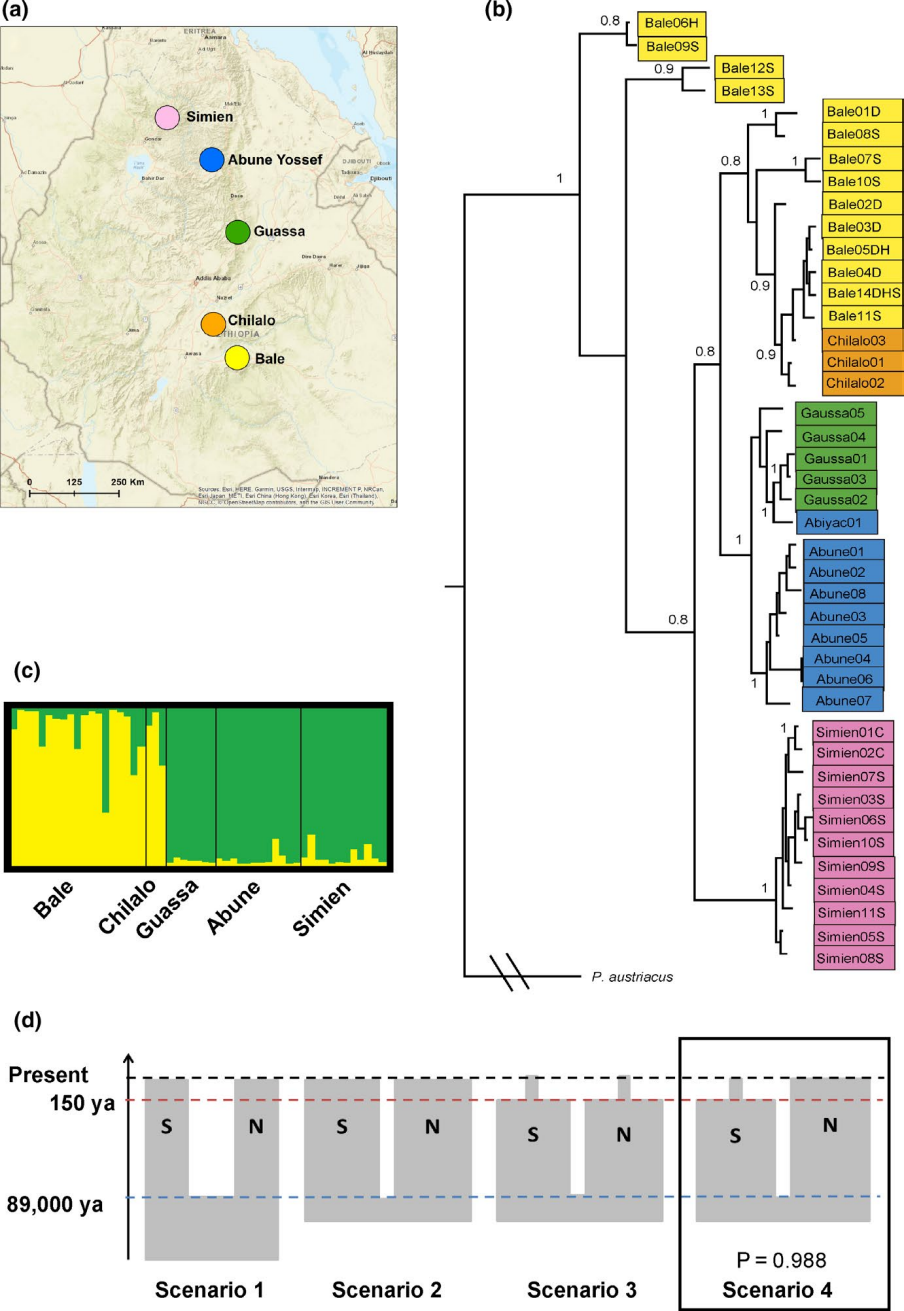
*Plecotus balensis* genetic population structure: (a) colour‐coded mountain ranges sampled in the study; (b) Bayesian phylogenetic tree based on the combined cytb and HVI mtDNA regions, with *Plecotus austriacus* as an outgroup and showing branch support > 0.8; (c) results of the STRUCTURE analysis dividing the microsatellite dataset into two population clusters, south (yellow) and north (green) of the Rift Valley; and (d) the best‐supported scenario in the approximate Bayesian computation analysis of demographic history (scenario 4), with width of boxes reflecting relative population sizes, S and N denoting south and north of Rift Valley populations, blue dashed line for population split time, red for recent population decline and black for the present time

We identified 32 unique cytb haplotypes and 34 unique HVI haplotypes (GenBank Accession numbers to be added after acceptance). Each mountain range had unique haplotypes and no haplotypes were shared between mountain ranges. Overall, the Bayesian phylogenetic tree showed a strong effect of mountain ranges on genetic population structure and supported a split between haplotypes to the north‐west and south‐east of the Rift Valley. The main divergence was identified between haplotypes from Bale [Harenna Forest and Sanetti Plateau (Bale‐S)] and the remaining haplotypes, followed by the divergence of Simien haplotypes. Only later haplotypes were split between north‐west and south‐east of the Rift Valley (Figure 2b). Population structure analysis of the microsatellite dataset split individuals into two clusters (K = 2), north and south of the Rift Valley (Figures [Link], [Link]). There were no further splits within each cluster. There was some evidence of shared ancestry between both sides of the Rift Valley (Figure 2c). F_ST_ and Jost's D values were strongly correlated (MRDM: *R* ^2^ = 0.684, *p* = .018). F_ST_ values confirmed the separation between populations north and south of the Rift Valley, with low values between the two Bale populations south of the valley and highest values between populations on either side of the Rift Valley (Table S5).

Based on the mtDNA dataset, the Bale‐S population had highest nucleotide diversity, substantially higher than the rest of the populations. The Simien population had lowest nucleotide diversity, but the highest haplotype diversity, though differences in haplotype diversity between populations were negligible (Table 2). Based on the microsatellite dataset, all populations had relatively high and similar levels of genetic diversity, with a particularly high number of private alleles identified in the Guassa population. Abune Yosef and Bale‐S had the highest levels of inbreeding, while Simien and Guassa had the lowest, though all values were low (Table 2).

**Table 2:** Population genetics summary statistics for *Plecotus balensis*. Columns 3–6 include mean number of alleles (Na), allelic richness corrected for sample size (Ar), number of private alleles (Pa) and average inbreeding (TrioML) per population for the microsatellite dataset. Columns 7–9 show number of haplotypes (Hap), haplotype diversity (Hd) and nucleotide diversity (Pi) per population for the mitochondrial DNA (concatenated Cytb and HVI sequences) dataset. (*N* = sample size)

Population	*N*	Na	Ar	Pa	TrioML	Hap	Hd	Pi
Bale‐D	7	5.579	5.286	11	0.066	6	0.952	0.0065
Bale‐S	9	6.737	5.694	17	0.081	8	0.972	0.0297
Guassa	7	6.368	5.139	26	0.055	5	0.857	0.0061
Simien	12	6.105	5.605	16	0.051	11	0.985	0.0029
Abune	11	6.632	5.907	14	0.096	8	0.972	0.0065

Allelic richness corrected for sample size decreased as the proportion of arable land in 5 km radius around the population capture location increased (*F* = 10.41, *df* = 1,3, *p* = .048, *R* ^2^ = 0.717; Figure S5). Levels of inbreeding did not relate to any of the land‐use variables (Table S6).

### 3.3 Landscape barriers to gene flow

The topographic hypothesis (effect of altitude) had the strongest support (AICcmin = 0.477, BICew = 0.351), followed by the ecoregions hypothesis (AICcmin = 0.412, BICew = 0.303). Confidence intervals of both variables did not overlap zero, supporting their effect on gene flow (Table 3). Topography and ecoregions were also the best‐supported models based on Jost's D, however, the ecoregions model was ranked higher than altitude (Table S7). Projected movement density maps based on the effect of these two landscape variables highlight the strong effect of the Rift Valley on genetic connectivity in *P. balensis* and the split between populations to the north and south of the valley (Figure S6). The remaining hypotheses had very low support. Hypotheses that included multiple variables had little support, likely due to the small number of populations (Table 3).

**Table 3:** Results of the MLPE landscape genetics analysis (based on F_ST_ measure of genetic differentiation), listing the hypotheses tested, the variables included in each model, model support based on AICc and BIC and evidence weights of each model (AICcmin and BICew) and the 95% confidence intervals of variables in the two best‐supported models

Hypothesis	Variables	AICc	BIC	AICcmin	BICew	95% CI
**Topography**	**Altitude**	**−85.884**	**−92.673**	**0.477**	**0.351**	**0.005–0.011**
**Ecoregions**	**Ecoregions 4**	**−85.589**	**−92.379**	**0.412**	**0.303**	**0.003–0.006**
Hydrology	Streams 2a	−81.253	−88.042	0.047	0.035	
Land cover	Land cover 4	−79.852	−86.642	0.023	0.017	
Forest	Per cent tree cover	−79.401	−86.191	0.019	0.014	
Anthropogenic	Human footprint	−77.820	−84.610	0.008	0.006	
Anthro‐Topo	Human + Altitude	−77.343	−90.831	0.007	0.140	
Anthro‐Eco	Human + Ecoregions	−76.591	−90.078	0.005	0.096	
Hydro‐Land	Land cover + Streams	−74.705	−88.193	0.002	0.037	

Best supported models highlighted in bold.

### 3.4 Demographic history

The best‐supported scenario was of a recent approximately six‐fold decline of the south‐eastern population but no change in the north‐western population (scenario 4; probability of scenario 0.988, overall model error < 0.001, type 1 error = 0.095, type 2 error averaged over scenarios (±standard deviation) = 0.046 ± 0.04; Figure S7; Table S8). Model checking for the most probable scenario (scenario 4) indicated a good fit between observed and simulated datasets, whereby the observed dataset fell within the cloud of simulated points (Figure S8). Rift Valley split time was estimated at a median of 89,600 years ago (95% CI: 35,000–155,400), based on a generation time of 2 years, while the decline of the south‐eastern population was estimated to have occurred at a median of 150 years ago (95% CI: 30–1000; Figure 2d). Bias in parameter estimation was low (mean relative bias 0.18–0.26 for population size parameters, and 0.29 for time of decline of southern population; Table S9).

## 4 DISCUSSION

Assessing threats to biodiversity under global change requires an understanding of the effects of past, current and future environmental changes on species distribution, abundance and genetic composition. Through combining genetic, ecological and geographical information with ecological niche and evolutionary history modelling approaches we show how historic climatic changes during the Quaternary have shaped the population structure and patterns of genetic variation of the Ethiopian Highlands long‐eared bat. We further show the impact of recent anthropogenic land‐use change and habitat degradation on population declines and loss of genetic diversity, and warn of threats due to disappearing suitable niche under future climate change. Our findings have important implications for biodiversity conservation in Afromontane and Afroalpine ecoregion more generally, which represent some of the most endemism rich and endangered ecosystem in the world (Williams et al., 2004).

### 4.1 Effects of climate change past and future

Ecological niche models support the restricted distribution of *P. balensis* in Afroalpine moorland and Afromontane grassland and woodland high elevation regions of the Ethiopian highlands, and show that its distribution is primarily limited by temperatures. Models projected across temporal scales show that climatic suitability for *P. balensis* has been progressively decreasing since the LGM and will continue to decrease with increasing temperatures over the next few decades, with a direct effect on population fragmentation and isolation. The genus *Plecotus* is of Palearctic origin and is found primarily across temperate and Mediterranean habitats (Spitzenberger et al., 2006). As such, it is not surprising that climatic conditions in the tropics were more suitable for this bat when temperatures were lower during the LGM. Increased climatic suitability during LGM and displacement of the Afroalpine and ericaceous zone to lower elevations (Bonnefille et al., 1990) allowed high‐altitude species to extend their range (Gottelli et al., 2004) and likely led to contact between isolated sky island populations (McCormack et al., 2009). This can explain the observed limited differentiation between mountain ranges not separated by the substantially lower Rift Valley.

Sky islands are sensitive to future climate change because the high montane and alpine habitats could contract into higher elevations with even minor temperature increases, reducing the habitat available for their associated endemic taxa (McCormack et al., 2009). Tropical montane forests, in particular, are some of the most threatened habitats under climate warming (Moritz & Agudo, 2013). Tropical montane regions are predicted to experience highest levels of disappearing climates, and consequently species extinctions and community disruptions (Williams et al., 2007). In Ethiopia, Nyssen et al. (2014) already identified evidence of upper shifts of the high elevation treeline in response to climatic changes in the past century. This ecotone is located between the Afromontane and Afroalpine zones and is particularly rich in biodiversity (Kidane et al., 2012). Indeed, our models predict that only a quarter of the current range of *P. balensis* will remain climatically suitable by the end of the century. These predictions are relevant to other high‐altitude Afromontane biodiversity, and therefore are particularly worrying given the high levels of endemism found in this ecoregion, in particular among vertebrates (Williams et al., 2004) and vascular plants (Gizaw et al., 2016).

### 4.2 Effects of geographical barriers

The Ethiopian Rift Valley was identified as the main barrier to contemporary gene flow shaping the population structure of *P. balensis*. The Great Rift Valley played a major role in structuring biodiversity across Eastern Africa, from the Afroalpine plants *Arabis alpine* (Asefa et al., 2007) and *Lobelia giberroa* (Kebede et al., 2007) to most of Ethiopian anurans (Freilich et al., 2016). As *P. balensis* is associated with low temperatures and montane ecosystems above 2,000 m (Benda et al., 2004), the much warmer and drier savannah‐like habitats at low elevation along the Rift Valley are very likely to be inhospitable for this bat. In fact, according to our data, the main split along the Rift Valley, can be traced to the last interglacial period when conditions were even warmer and drier than in the Holocene (Adams et al., 1999).

The complex geological history of the Ethiopian highlands is partially responsible for its high biodiversity and endemism. The highlands do not constitute a single unit, but instead are formed by various massifs of different areas, origins, ages and degrees of isolation (Reyes‐Velasco et al., 2018). This geographical and historical complexity is expected to affect the population structure of *P. balensis*. A signature of population isolation in sky islands is seen at the mtDNA dataset where no haplotypes were shared between mountain ranges and all mountain ranges formed separate clades in the phylogenetic tree, although all were nested within the deeply divergent basal lineages formed of four Bale haplotypes. Mountain range separation could be the result of isolation processes relating to older glacial cycles during the Pleistocene. These episodes of allopatric isolation appear as the main driver of differentiation in Ethiopian highland vertebrates (Reyes‐Velasco et al., 2018). The phylogeographic structure of *P. balensis* is consistent with that of the Ethiopian wolf (Gottelli et al., 2004), whereby three main clusters are present corresponding to the three well‐defined mountain areas: the southern Chilalo/Bale, the central Guassa/Abune Yosef and the northern isolated Siemen Massifs. This strong structure at the mtDNA level indicates that sky islands have acted as historic barriers to gene flow in *P. balensis*.

More limited population structure based on bi‐parentally inherited markers suggests that gene flow between mountain ranges is primarily male‐mediated, a common pattern in temperate bat species (Moussy et al., 2013). However, as extensive contemporary gene flow between mountain ranges is unlikely given the extent of habitat conversion across the Ethiopian highlands (Lemenih & Kassa, 2014), the shallow genetic structure at the nuclear level, may instead indicate habitat connectivity during the Holocene, possibly through montane forest bridges during episodes of increased moisture (Umer et al., 2007). Alternatively, genetic differentiation may be driven by local adaptations and environmental dissimilarity and therefore its signature cannot be identified in neutral markers (Manthey & Moyle, 2015).

### 4.3 Effects of anthropogenic land‐use change

Although ENMs identified that *P. balensis* is primarily associated with Afroalpine moorland and grassland and Afromontane woodland ecoregions, reproductive females and sub‐adults were only found in high‐altitude Afromontane woodlands, highlighting the importance of this habitat for the species’ reproductive success. A similar pattern of elevational segregation was observed in other Palearctic bats, such as the congeneric alpine long‐eared bat *Plecotus macrobullaris*, whereby maternity colonies in colder regions of the distribution are found below the treeline. Alberdi et al. (2014) attribute this segregation to restrictions on facultative heterothermy and use of torpor in pregnant and lactating females.

The Afromontane woodland habitat is highly threatened and disappearing due to extensive deforestation and overgrazing (Yalden et al., 1996). Deforestation rates in the Ethiopian Highlands are severe following centuries of landscape changes due to subsistence farming, settlement and demands for fuelwood (Lemenih & Kassa, 2014). Native forest cover has declined in the past 100 years from 45% to only 5% of the country, and currently most of the native forest cover in the highlands is concentrated in small patches surrounding orthodox churches, known as church forests (Abbott, 2019). The mountain forest belt has been pushed upwards and fragmented due to extensive agriculture (Kebede et al., 2007). Some of the forests where bats were caught south of the Rift Valley were degraded, fragmented and showed limited evidence of recruitment due to overgrazing of ground vegetation cover. This represents a broader trend of forest degradation in the Bale Mountains region, including inside the National Park, due to logging, fuelwood collection and livestock grazing (Asefa et al., 2015), which has been linked with the recent declines of most large wild mammals populations within the park (Stephens et al., 2001).

The recent decline of the south‐eastern *P. balensis* population identified in our ABC model‐based inference appears to coincide with a period of accelerated forest loss and degradation and the expansion of agriculture, though causal mechanisms behind the decline were not tested here. No evidence of decline in the north‐western population could be attributed to the general higher density of montane forests in the wetter north, as well as the buffering effect of the thousands of church forests containing native trees that are spread across the north‐western highlands (Klepeis et al., 2016). However, small sample sizes may have limited the inference power of the ABC analysis when it comes to identifying more moderate bottlenecks. Genetic diversity in the Ethiopian wolf was also higher in the northern highlands (Gottelli et al., 2004), indicating that habitat degradation in the south is having a detrimental effect on Afroalpine and Afromontane mammals in general. Although native forest loss has been more limited in the northern highlands over the past 150 years, degradation is ongoing in the more recently settled Simien mountains (Nyssen et al., 2014) and Abune Yosef Conservation Area has seen recent development of settlements and roads due to limited legal protection. Therefore, the northern highlands Afromontane biodiversity is also at risk.

Despite the strong effect of anthropogenic land‐use change on genetic diversity and population decline in *P. balensis*, indicators of anthropogenic impact, like artificial lights at night and human footprint index, did not affect gene flow. Low sample size (five populations) may have reduced the power of the landscape genetics statistical analysis, in particular when considering the combined impact of more than one variable, and therefore this limitation may have obscured the importance of some drivers of landscape resistance. Although native church forest patches and small community‐managed forest reserves offer some level of landscape connectivity across the agricultural and human settlement landscape, per cent tree cover did not affect genetic connectivity. However, this may be attributed to the tree cover map used, which does not distinguish between native forests and eucalyptus plantations (Hansen et al., 2013), and therefore could not capture the effect of the landscape on the movement of *P. balensis*.

The landscape genetics analysis highlights the importance of altitude and Afroalpine and Afromontane habitats. The best‐supported model gave lowest resistance costs to the high‐altitude alpine moorland ecoregion, rather than the montane forest ecoregion, perhaps due to the more restricted distribution of native montane forest than the ecoregion classification suggests because of extensive deforestation in the past century. Alternatively, this may reflect temperature or other climatic limitations on the movement of *P. balensis*, as is the case with other cold‐adapted high‐altitude mammals, like the American pika, *Ochotona princeps* (Castillo et al., 2014). Altitude was also identified as the main landscape element limiting gene flow in high‐altitude salamanders, alongside pond network structure. However, genetic connectivity in these more limited dispersal species was primarily affected by geographic distance presumably due to the rarity of dispersal events in the extreme high‐altitude environment (Savage et al., 2010).

## 5 CONCLUSIONS

Using a combination of ecological niche models, landscape genetics and ABC model‐based inference of demographic history, we show how historic climate change and geographic barriers interact with recent anthropogenic habitat loss and degradation to shape the population size, structure, diversity and connectivity of tropical montane biodiversity. Focusing on the endemic Ethiopian Highlands bat, *P. balensis*, we found that despite strong associations with high‐altitude environments and mtDNA pattern associated with sky island structure, some level of genetic connectivity is maintained among sky islands, and only substantially lower altitudes, like the Rift Valley, form a true barrier to gene flow. Of particular concern are evidence of recent population decline, likely in response to deforestation and land conversion, the decline in genetic diversity with increasing arable land cover, and the importance of Afroalpine and Afromontane ecoregions for both range suitability and genetic connectivity between sky islands. Given that similar patterns of genetic population structure have been recorded in other high‐altitude mammals and amphibians, such losses of genetic diversity and population declines are likely a widespread ongoing process in tropical montane ecosystems. The situation described for this bat epitomizes a wider trend in high‐altitude Afromontane and Afroalpine biodiversity, which is under threat from extensive deforestation, agricultural conversion and overgrazing following rapid human population expansion (Yalden et al., 1996). These threats are not likely to be reversed in the future in countries like Ethiopia where accelerated human population growth is projected to continue until the end of the century (UN, 2019). These immediate threats will interact with future climate change and the projected disappearance of suitable climates and their associated ecoregions in tropical montane regions (Williams et al., 2007), which will increase the likelihood of extinction of high‐altitude species. This study presents alarming predictions for the fate of tropical montane and alpine biodiversity, projected to be squeezed to higher altitudes (or even disappear) due to climate change while already losing genetic diversity and suffering population declines due to anthropogenic land‐use change. The combination of these different threats can push these ecosystems beyond their resilience limits. We conclude that assessments of threats to biodiversity under global change should adopt a holistic approach, simultaneously studying the effects of multiple threats and considering the impacts of past events, present stressors and future projections based on genetic, ecological and geographic information.

## CONFLICT OF INTEREST

None declared.

## AUTHOR CONTRIBUTIONS

OR and JJ conceived and designed the study. OR, MK, HS and JJ collected the samples. OR and JJ carried out the laboratory work. OR performed the data analysis and modelling. OR and JJ wrote the manuscript, and all authors contributed to revisions.

## Supporting information

AppendixA

AppendixB

## Data Availability

DNA sequences were submitted to GenBank. Cytochrome b Accession numbers: MW166383‐MW166433. Control Region HV1 accession number: MW166434‐MW166484. Microsatellite dataset, mtDNA sequence assembly, Maxent output files and STRUCTURE results are made available through Dryad (https://doi.org/10.5061/dryad.k3j9kd55n). Sampling locations are uploaded as Appendix B Supporting Table S1.
