# Supplementary material for: Up in the air: Threats to Afromontane biodiversity from climate change and habitat loss revealed by genetic monitoring of the Ethiopian Highlands bat
Source: Evol Appl. 2020 Dec 7;14(3):794–806. doi: 10.1111/eva.13161 (PMC7980307; doi:10.1111/eva.13161)
Supplement: Supplementary file 1 — AppendixA [file EVA-14-794-s001.pdf]

Appendix A – Supporting Methods

Up in the air: threats to Afromontane biodiversity from climate change and habitat loss revealed by genetic monitoring of the Ethiopian Highlands bat

Ecological niche modelling

The full model included a combination of climatic [downloaded from WorldClim (www.worldclim.org/)], topographic [including altitude, slope and ruggedness, calculated from SRTM map (https://www2.jpl.nasa.gov/srtm/); landscape ruggedness was calculated from the altitude map by computing the maximum elevation difference within a 5 km buffer around each cell using range statistics in ArcGIS v10.3.1], land cover [GlobCover2009 map, European Space Agency (http://due.esrin.esa.int/page_globcover.php) reclassified into 10 general categories], vegetation cover [Normalized Difference Vegetation Index (NDVI) for the dry and wet seasons, MOD13A3 (https://lpdaac.usgs.gov/)], ecoregions (WWF ecoregions map; Olson et al., 2001), and human footprint variables [Global human footprint v2 2005, NASA (http://sedac.ciesin.columbia.edu/data/set/wildareas-v2-human-footprint-geographic)].

Generating the genetic dataset

For mitochondrial DNA markers, the PCR reaction (20µl final reaction volume) included 0.5-5µl of DNA extract, 0.5µM of each primer, 2nM of $MgCl_2$, 0.2nM of dNTPs, 0.2mg BSA, and 0.5 units of Taq-Polimerase. Thermo-cycling consisted of 4 min initial denaturation at 94ºC followed by 35 cycles of 60s at 94ºC, 30s at 52ºC (for both pairs) and 90s at 72ºC and a final extension of 10 min at 72ºC. The PCR products were sequenced using ABI 3100 automated

sequencers (PE Biosystems, Warrington, UK) and DNA fragments were aligned and edited using Geneious v. R11 software (Biomatters Ltd).

For microsatellite markers, the forward primer of each locus pair was labelled fluorescently with HEX or 6-FAM (Applied Biosystems), and microsatellites were combined into six multiplex sets. Each 10µl PCR plex contained 2–4 primer sets (each set at a concentration of 0.2µM and a total volume of 1µl), 5µl Qiagen multiplex PCR master mix and 2µl DNA. We used the following PCR program: initial denaturation at 95°C for 15 min, followed by 35 cycles of 94°C for 30s, 57°C for 90s and 72°C for 60s, followed by a final extension at 60°C for 30 min. PCR products were sent for fragment analysis in Dnaseq (University of Dundee, UK). Allele sizes were assigned using GeneMapper (Applied Biosystems, USA).

Power analysis

We tested the power of our genetic dataset to detect population structure (assignment tests and connectivity) and identify a population bottleneck using the Sample Planning Optimization Tool for conservation and population Genetics (SPOTG; Hoban, Gaggiotti, & Bertorelle, 2013). Assignment tests had very high power (0.98) with 20 individuals and two populations and high power with 10 individuals and five populations (0.86). Similarly, connectivity analysis had high power (0.87) even with our minimum population size (7 individuals), while for 10 individuals, power has increased to very high (0.97). The power to detect a recent bottleneck varied depending on the extent of the bottleneck and sample size. For a severe bottleneck, a sample size of 20 individuals had high power (79%), while 10 individuals had insufficient power (27%). For a moderate bottleneck, an analysis with 20 individuals had just below minimal chance of success (46%), and 10 individuals had very low power (20%).

Supplementary References

Hoban, S., Gaggiotti, O., Bertorelle, G., 2013. Sample Planning Optimization Tool for conservation and population Genetics (SPOTG): a software for choosing the appropriate number of markers and samples. Methods Ecol. Evol. 4, 299–303.

Olson, D.M., Dinerstein, E., Wikramanayake, E.D., Burgess, N.D., Powell, G.V.N., Underwood, E.C., D'amico, J.A., Itoua, I., Strand, H.E., Morrison, J.C., Loucks, C.J., Allnutt, T.F., Ricketts, T.H., Kura, Y., Lamoreux, J.F., Wettengel, W.W., Hedao, P., Kassem, K.R., 2001. Terrestrial Ecoregions of the World: A New Map of Life on Earth. Bioscience 51, 933–938.
