# Supplementary material for: Up in the air: Threats to Afromontane biodiversity from climate change and habitat loss revealed by genetic monitoring of the Ethiopian Highlands bat
Source: Evol Appl. 2020 Dec 7;14(3):794–806. doi: 10.1111/eva.13161 (PMC7980307; doi:10.1111/eva.13161)
Supplement: Supplementary file 2 — AppendixB [file EVA-14-794-s002.pdf]

Appendix B – Supporting Tables and Figures

Up in the air: threats to Afromontane biodiversity from climate change and habitat loss revealed by genetic monitoring of the Ethiopian Highlands bat

Table S1 – List of sample collection locations, their associated population and number of males and females caught in each site.

Mountain range	Population	Location	Habitat	Latitude	Longitude	Altitude (m)	Date	Males	Female	Total
Bale Mountains	Bale-D	Dinsho	Juniper forest	7.09445	39.79046	3195	Oct 2014	3	1	4
Bale Mountains	Bale-D	Gesse forest, Dinsho	Juniper forest	7.10681	39.73461	3237	Oct 2014	2	0	2
Bale Mountains	Bale-D	Gesse forest, Dinsho	Juniper forest	7.10544	39.73736	3237	Oct 2014	1	0	1
Bale Mountains		Harenna forest, Rira	Afromontane forest	6.75296	39.71883	2795	Oct 2014	1	1	2
Bale Mountains		Harenna forest, Rira	Afromontane forest	6.75229	39.71394	2800	Oct 2014	0	1	1
Bale Mountains	Bale-S	Sanetti Plateau, campsite	Afroalpine grasslands	6.85594	39.88123	4007	Oct 2014	2	0	2
Bale Mountains	Bale-S	Sanetti Plateau, Red cliff	Afroalpine grasslands	6.80448	39.80022	3918	Oct 2014	7	0	7
Chilallo-Galame		Asella, Arsi	Juniper forest /arable	7.95669	39.18901	2954	Oct 2014	2	0	2
Chilallo-Galame		Western slopes, Arsi	Juniper forest /arable	7.93639	39.19770	3165	Dec 2015	1	0	1
Simien Mountains	Simien	Chenoke camp	Afroalpine grasslands	13.26377	38.20847	3949	April 2015	1	0	1
Simien Mountains	Simien	Chenoke camp	Afroalpine grasslands	13.22337	38.22074	4224	April 2015	1	0	1
Simien Mountains	Simien	Sankaber camp	Erica forest	13.23111	38.03075	3192	April 2015	4	6	10
Abune-Yosef	Abune	Abune Yosef, cliff camp	Afroalpine grasslands	12.14512	39.17923	4155	April 2015	2	0	2
Abune-Yosef	Abune	Abune Yosef, Zigit	Afroalpine grasslands	12.15794	39.15246	3773	April 2015	1	0	1
Abune-Yosef	Abune	Abune Yosef, pass	Afroalpine grasslands	12.15323	39.15316	3849	April 2015	6	0	6
Guassa	Guassa	Guassa CCA, lodge1	Erica forest	10.31139	39.80500	3492	Sep 2015	1	0	1
Guassa	Guassa	Guassa CCA, lodge2	Erica forest	10.30278	39.80861	3378	Sep 2015	1	0	1
Guassa	Guassa	Guassa CCA, Legora	Afromontane grass	10.40806	39.80717	3150	Sep 2015	1	1	2
Guassa	Guassa	Guassa CCA, pass	Afromontane grass	10.40805	39.80722	3358	Sep 2015	2	1	3
**TOTAL**								39	11	50

Table S2 – List of variables included in the final full *Plecotus balensis* ecological niche model and their percent contribution to the model. Asterix mark variables included in the climatic models.

**Variable**	**Source**	**Contribution to model**
Maximum temperature (BIO5)*	WorldClim (www.worldclim.org/)	57.1%
Ecoregions	WWF (Olson et al. 2001)	33.4%
Topographic ruggedness*	SRTM map (www2.jpl.nasa.gov/srtm/)	6.7%
Land cover	GlobCover2009 map (http://due.esrin.esa.int/page_globcover.php)	2.8%

Table S3 – Landscape variables included in the landscape genetics analysis with their source maps, effect on movement, with justification in brackets, and assigned resistance costs.

Variable	Source	Effect on movement [justification]	Resistance costs
Altitude	SRTM map (www2.jpl.nasa.gov/srtm/)	Resistance to movement decreases with altitude [only found in high altitudes – above 3000 masl]	1-100 (high-low altitudes)
Altitude 10q	SRTM map (www2.jpl.nasa.gov/srtm/)	Resistance decreases with altitude	10 categories classified based on 10 quantiles (costs increase in 10 intervals)
Altitude 10g	SRTM map (www2.jpl.nasa.gov/srtm/)	Resistance decreases with altitude	10 categories classified based on geometric intervals (costs increase in 10 intervals)
Altitude 3 a/b/c	SRTM map (www2.jpl.nasa.gov/srtm/)	No resistance at altitudes where found, maximum resistance at Rift Valley altitudes	3 categories:<br>> 3000m = 1<br>2000-3000m = 10 / 20 / 50<br>< 2000m = 100
Topographic ruggedness	SRTM map (www2.jpl.nasa.gov/srtm/)	Resistance decreases with ruggedness [found in areas with high topographic ruggedness – based on ENM]	1-100 (high-low ruggedness)
Ruggedness 10q	SRTM map (www2.jpl.nasa.gov/srtm/)	Resistance decreases with ruggedness	10 categories classified based on 10 quantiles (costs increase in 10 intervals)
Ruggedness 10g	SRTM map (www2.jpl.nasa.gov/srtm/)	Resistance decreases with ruggedness	10 categories classified based on geometric intervals (costs increase in 10 intervals)
Ecoregions 4	WWF (Olson et al. 2001)	High resistance to movement in non-montane ecoregions [only found in montane habitats]	4 categories:<br>Et mont moorland =1<br>Et mont grass/wood = 1<br>Et mont forest = 10<br>all the rest = 100
Ecoregions 5	WWF (Olson et al. 2001)	Higher resistance to movement in non-montane ecoregions	5 categories:<br>Et mont moorland = 1<br>Et mont grass/wood = 10<br>Et mont forest = 20<br>Som mont wood = 50<br>wood/savanna = 70<br>all the rest = 100

Variable	Source	Effect on movement [justification]	Resistance costs
Ecoregions 6	WWF (Olson et al. 2001)	Higher resistance to movement in non-montane ecoregions	6 categories:<br>Et mont moorland = 1<br>Et mont grass/wood = 1<br>Et mont forest = 10<br>Som mont wood = 20<br>wood = 30<br>savannah = 50<br>all the rest = 100
Ecoregions 7	WWF (Olson et al. 2001)	Higher resistance to movement in non-montane ecoregions	6 categories:<br>Et mont moorland = 1<br>Et mont grass/wood = 10<br>Et mont forest = 20<br>Som mont wood = 30<br>wood = 40<br>savannah = 60<br>all the rest = 100
Vegetation cover (NDVI)	MOD13A3 (https://lpdaac.usgs.gov/)	Resistance to movement decreases with dry season vegetation cover [slow flying bat will likely require vegetation cover to hide from predators when flying]	1-100 (high-low NDVI)
Land cover 4	GlobCover2009 map (http://due.esrin.esa.int/page_globcover.php)	Resistance to movement increases with anthropogenic impact [categories defined based on areas where the species was successfully captured]	4 categories:<br>forest / mosaic forest-grass = 1<br>mosaic crop-forest = 10<br>shrub / grass / crop = 50<br>urban / barren = 100
Land cover 7	GlobCover2009 map (http://due.esrin.esa.int/page_globcover.php)	Resistance to movement increases with anthropogenic impact	7 categories:<br>mosaic forest-grass = 1<br>forest = 10<br>shrub = 20<br>mosaic crop-forest = 30<br>grass / crop = 60<br>water = 70<br>urban / barren = 100
Percent tree cover	(https://earthenginepartners.appspot.com/science-2013-global-forest/download_v1.2.html)	Resistance to movement decreases with tree cover<br>[Slow flying bats - trees provide cover from predators when flying]	1-100 (high-low tree cover)

Variable	Source	Effect on movement [justification]	Resistance costs
Percent tree cover 3 a/b/c	(https://earthenginepartners.appspot.com/science-2013-global-forest/download_v1.2.html)	Resistance to movement increases when tree cover very low	3 categories:<br>51-99% cover = 1<br>16-50% cover = 10/ 20/ 50<br>0-15% cover = 100
Distance to streams	ESRI (www.arcgis.com/home/item.html?id=273980c20bc74f94ac96c7892ec15aff)	Resistance to movement increases with distance to permanent streams [These streams will have gallery forests, which will facilitate movement]	1-100 (low-high distance from streams)
Distance to streams 2 a/b	ESRI (www.arcgis.com/home/item.html?id=273980c20bc74f94ac96c7892ec15aff)	Resistance to movement increases with distance to permanent streams beyond likely gallery forest strip size	2 categories:<br>0 – 1 / 5 km = 1<br>> 1 / 5 km = 100
Human footprint	Global human footprint v2 (2005), NASA (http://sedac.ciesin.columbia.edu/data/set/wildareas-v2-human-footprint-geographic)	Resistance to movement increases with anthropogenic impact [These bats are not known to be found in urban area, and instead are associated with undisturbed habitats]	1-100 (low-high human footprint)
Night light	Night light development index (2006), NOAA (https://ngdc.noaa.gov/eog/dmsp/download_nldi.html)	Resistance to movement increases with anthropogenic impact	1-100 (low-high night lights)
Night light 10	Night light development index (2006), NOAA (https://ngdc.noaa.gov/eog/dmsp/download_nldi.html)	Resistance to movement increases with anthropogenic impact	10 categories classified based on 10 quantiles (costs increase in 10 intervals)

Table S4 – Results of MRDM tests to select the best combination of resistance costs for each variable group (hypothesis tested in the landscape genetic analysis) based on their relationship with genetic differentiation (F_{ST} or Jost's D) between *Plecotus balensis* populations. Selected variables are highlighted in green (for F_{ST}) or blue (for Jost's D).

		F_{ST}			Jost's D		
**Variable group**	**Layer**	**R^2**	**F**	**P**	**R^2**	**F**	**P**
Topographic	Abrupt	0.566	10.431	0.017	0.585	11.289	0.016
Topographic	Abrupt 10g	0.349	4.283	0.114	0.368	4.66	0.066
Topographic	Abrupt 10q	0.217	2.224	0.132	0.248	2.644	0.104
Topographic	Altitude	0.766	26.267	0.017	0.733	21.966	0.017
Topographic	Altitude 10g	0.679	16.943	0.019	0.631	13.657	0.035
Topographic	Altitude 10q	0.700	18.677	0.016	0.671	16.336	0.018
Topographic	Altitude 3a	0.721	20.688	0.019	0.665	15.892	0.031
Topographic	Altitude 3b	0.713	19.866	0.015	0.648	14.729	0.032
Topographic	Altitude 3c	0.697	18.373	0.018	0.621	13.121	0.031
Ecoregions	Ecoregions 4	0.827	38.261	0.014	0.705	19.107	0.018
Ecoregions	Ecoregions 5	0.807	33.382	0.015	0.679	16.908	0.017
Ecoregions	Ecoregions 6	0.636	13.959	0.017	0.649	14.848	0.017
Ecoregions	Ecoregions 7	0.796	31.241	0.017	0.666	15.977	0.017
Land cover	Land cover	0.475	7.241	0.053	0.358	4.462	0.085
Land cover	Land cover 4	0.641	14.306	0.017	0.523	8.776	0.037
Land cover	NDVI	0.457	9.744	0.082	0.619	13.003	0.032
Hydrology	Distance to streams	0.448	6.502	0.017	0.125	1.144	0.287
Hydrology	Streams 2a	0.639	14.159	0.354	0.692	17.984	0.018
Hydrology	Streams 2b	0.628	13.479	0.0322	0.644	14.477	0.017
Forest	tree cover	0.569	10.543	0.048	0.698	18.519	0.033
Forest	Tree cover 3a	0.225	2.328	0.149	0.273	2.998	0.113
Forest	Tree cover 3b	0.446	6.441	0.067	0.519	8.649	0.049
Forest	Tree cover 3c	0.143	1.335	0.235	0.181	1.773	0.187
Anthropogenic	Night lights	0.079	0.692	0.602	0.017	0.139	0.870
Anthropogenic	Night lights 10	0.085	0.745	0.562	0.020	0.167	0.848
Anthropogenic	Human footprint	0.480	7.383	0.066	0.801	32.31	0.016

Table S5 – Genetics differentiation (F_{ST}) between *Plecotus balensis* populations based on the microsatellite dataset, with F_{ST} in bottom triangle and Jost's D in top triangle.

	**Bale-D**	**Bale-S**	**Simien**	**Abune**	**Guassa**
**Bale-D**	0	0.013	0.267	0.221	0.275
**Bale-S**	0.003	0	0.259	0.175	0.243
**Simien**	0.078	0.074	0	0.124	0.189
**Abune**	0.059	0.046	0.035	0	0.103
**Guassa**	0.072	0.061	0.053	0.026	0

Table S6 – Results of the linear models relating allelic richness (corrected for sample size) and inbreeding (based on TrioML measure) in *Plecotus balensis* populations to land cover variables in 5 km radius around capture sites. Significant results highlighted in bold.

	**Allelic richness**				**Inbreeding**			
	**R^2**	**F**	**df**	**P**	**R^2**	**F**	**df**	**P**
NDVI dry	0.357	3.221	1,3	0.171	0.317	2.856	1,3	0.189
Human footprint	0.053	0.168	1,3	0.709	0.008	0.025	1,3	0.885
Forest	0.215	0.427	1,3	0.560	0.039	0.122	1,3	0.751
Arable	**0.717**	**10.41**	**1,3**	**0.048**	0.193	1.956	1,3	0.256

Table S7 – Results of the MLPE landscape genetics analysis based on Jost's D measure of genetic differentiation. Table lists the hypotheses tested, the variables included in each model, model support based on AICc and BIC and evidence weights of each model (AICcmin and BICew) and the 95% confidence intervals of variables in the two best supported models. The Hydro-Land hypothesis could not be included in the Jost's D analysis due to VIF values >4 between NDVI and streams.

Hypothesis	Variables	AICc	BIC	AICcmin	BICew	95% CI
**Topography**	**Altitude**	**-60.427**	**-67.216**	**0.254**	**0.196**	**0.015-0.036**
Land cover	NDVI	-53.557	-60.346	0.008	0.006	
Anthropogenic	Human footprint	-53.838	-60.628	0.009	0.007	
**Ecoregions**	**Ecoregions 4**	**-62.292**	**-69.082**	**0.645**	**0.499**	**0.009-0.021**
Forest	Tree cover	-55.502	-62.292	0.022	0.017	
Hydrology	Streams 2a	-57.224	-64.014	0.051	0.04	
Anthro-Eco	Human + Ecoregions	-53.359	-66.846	0.007	0.163	
Anthro-Topo	Human + Altitude	-51.702	-65.189	0.003	0.071	

Table S8 – Approximate Bayesian computation probability of scenario comparison based on the Logistic approach for different number of closest simulations to the observed data (# sims), showing the probability of each scenario (prob) and range of values (range) for the four tested scenarios.

# sims	s1 prob	s1 range	s2 prob	s2 range	s3 prob	s3 range	s4 prob	s4 range
8000	0	[0.000,0.000]	0.005	[0.000,0.025]	0	[0.000,0.000]	0.995	[0.975,1.000]
16000	0	[0.000,0.000]	0.006	[0.000,0.021]	0	[0.000,0.000]	0.994	[0.979,1.000]
24000	0	[0.000,0.000]	0.011	[0.000,0.036]	0	[0.000,0.000]	0.989	[0.964,1.000]
32000	0	[0.000,0.000]	0.012	[0.000,0.033]	0	[0.000,0.000]	0.988	[0.967,1.000]
40000	0	[0.000,0.000]	0.012	[0.000,0.031]	0	[0.000,0.000]	0.988	[0.969,1.000]

Table S9 – Parameter estimation based on the best-supported model (scenario 4) in the approximate Bayesian computation model-based inference of the demographic history of *Plecotus balensis* and results of the bias and precession on parameter estimation analysis (MR bias = mean relative bias; RMedAD = relative median absolute deviation). Smaller accuracy values correspond to more precise parameter estimation. See Supplementary Fig. S1 for scenario details.

Parameter	Median	Q5%	Q95%	MR bias	RMedAD
Na: current South population size	9.70E+03	8.52E+03	9.97E+03	0.18	0.31
N2: current North population size	9.20E+04	2.09E+04	5.71E+05	0.26	0.36
T4: time of South population decline	7.66E+01	1.48E+01	5.57E+02	0.29	0.42
N1a: past South population size after split	5.99E+04	2.38E+04	1.39E+05	0.20	0.33
T3: time of population split	4.48E+04	1.75E+04	7.77E+04	0.44	0.43

Supporting Figures

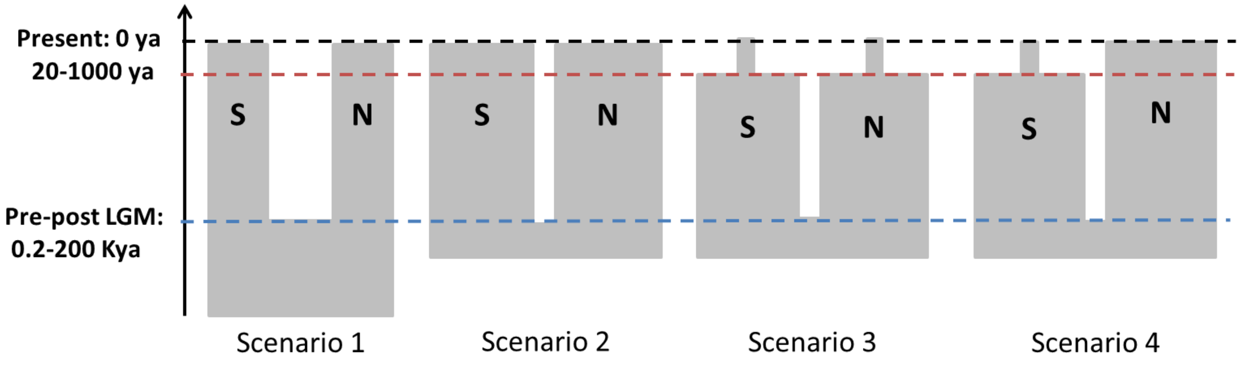

Figure S1 – The four scenarios compared in the approximate Bayesian computation model-based inference of *Plecotus balensis* demographic history. Width of boxes reflects relative population sizes. S and N denote South and North of Rift Valley populations, respectively. The three timeframes compared in the scenarios are indicated by the dashed lines: blue for population split time (priors set between 200 and 200,000 ya), orange for recent population size changes (priors set between 20 and 1,000 ya), and black for the present time (population sampling time, set as 0 ya).

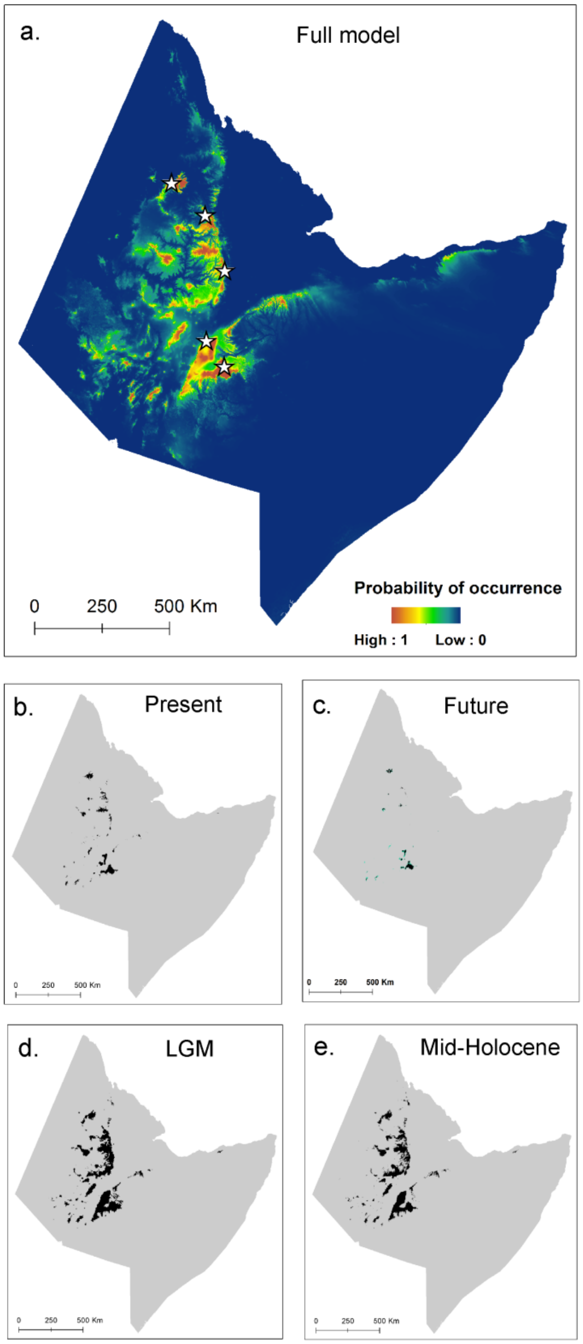

Fig. S2 – Projected probability of occurrence for *Plecotus balensis* across the horn of Africa based on ecological niche modelling outputs: a) based on the full model (probability of occurrence ranges from high in orange to low in blue; stars denote the location of mountain ranges sampled in this study); b) present climate model; c) future climate model (2070; RCP8.5 in black and RCP4.5 in blue); d) last glacial maximum (LGM) climate model; and e) mid-Holocene climate model. In the climate models black denote suitable areas and grey unsuitable

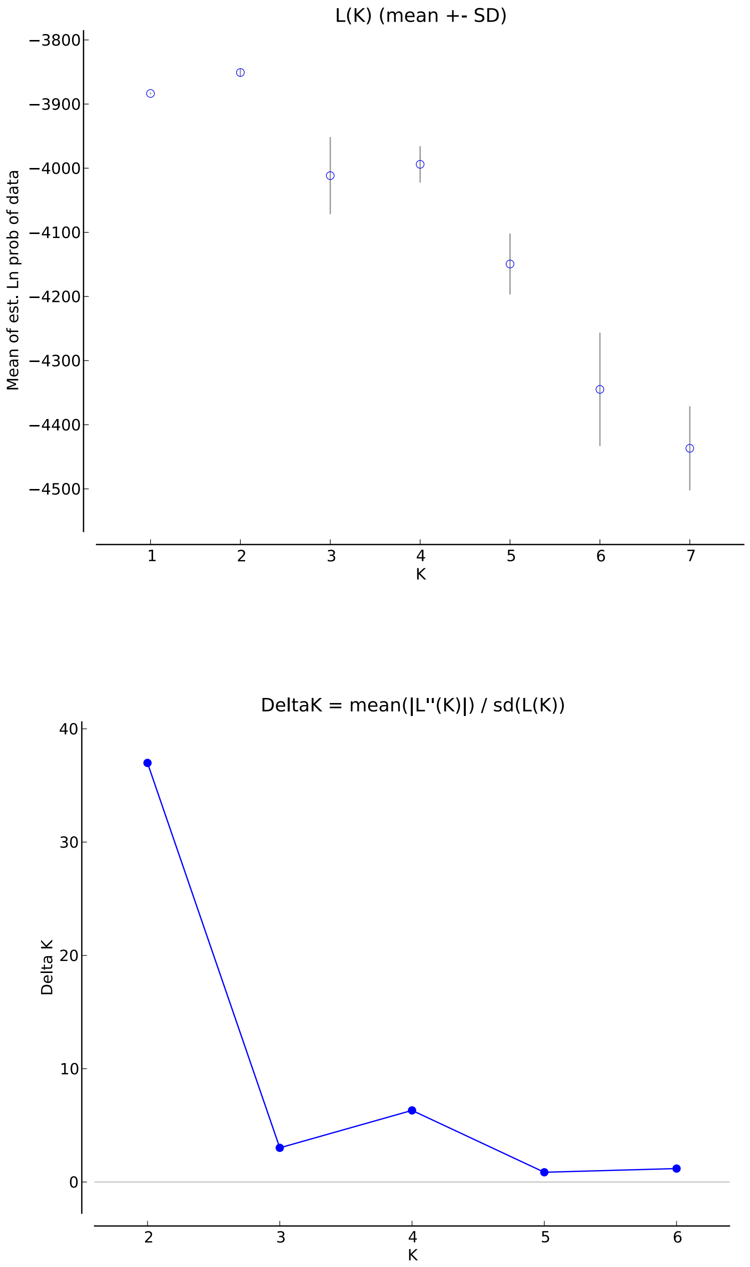

Fig. S3 – Results of the STRUCTURE analysis showing best supported number of clusters (K) based on the log Likelihood (top) and Evanno's delta K (bottom) methods. In both cases K=2 received the highest support.

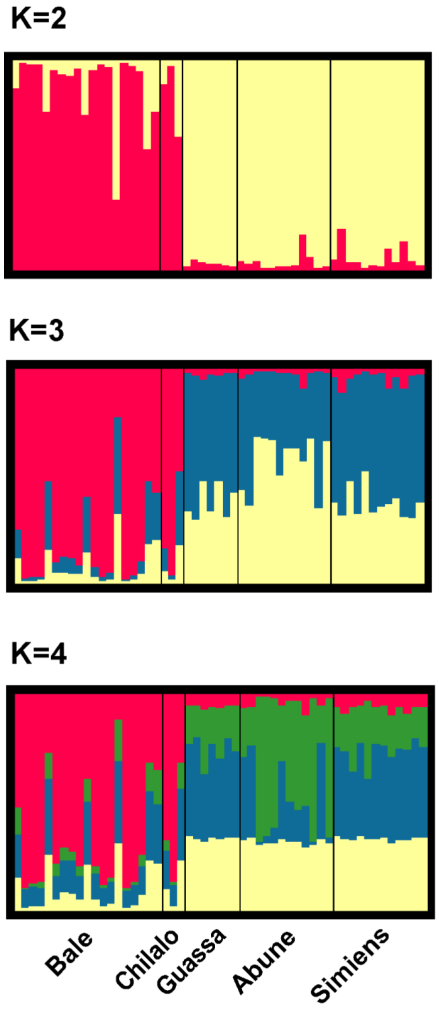

Figure S4 – Outputs of the Structure analysis showing ancestry of individuals for K = 2-4 population clusters.

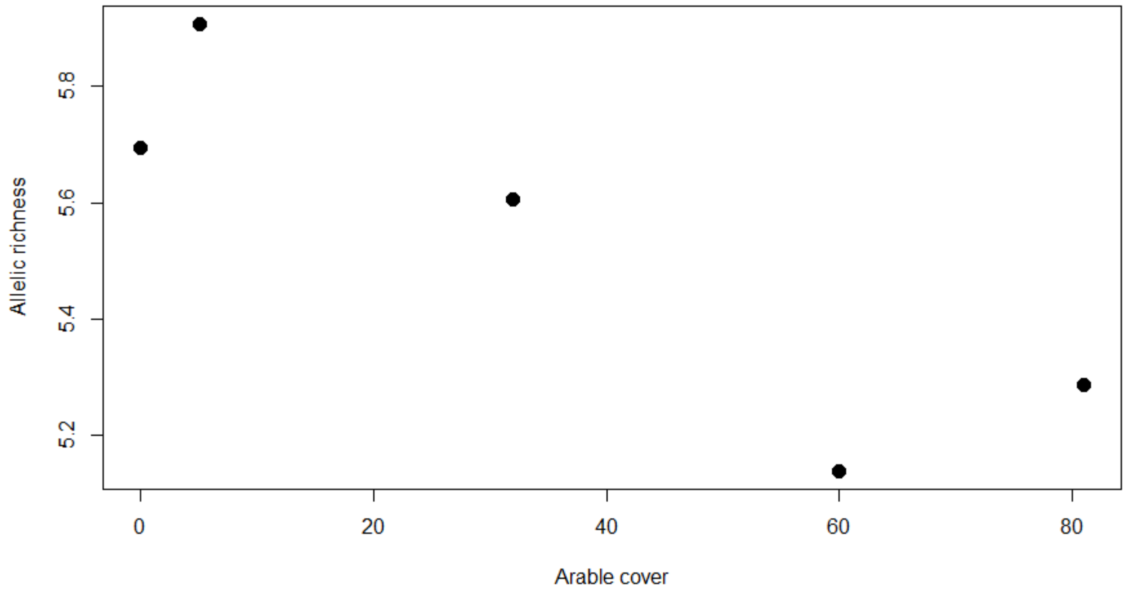

Figure S5 – The correlation between genetic diversity (allelic richness corrected for sample size) of *Plecotus balensis* populations and the proportion of arable land in 5 km radius around sampling sites.

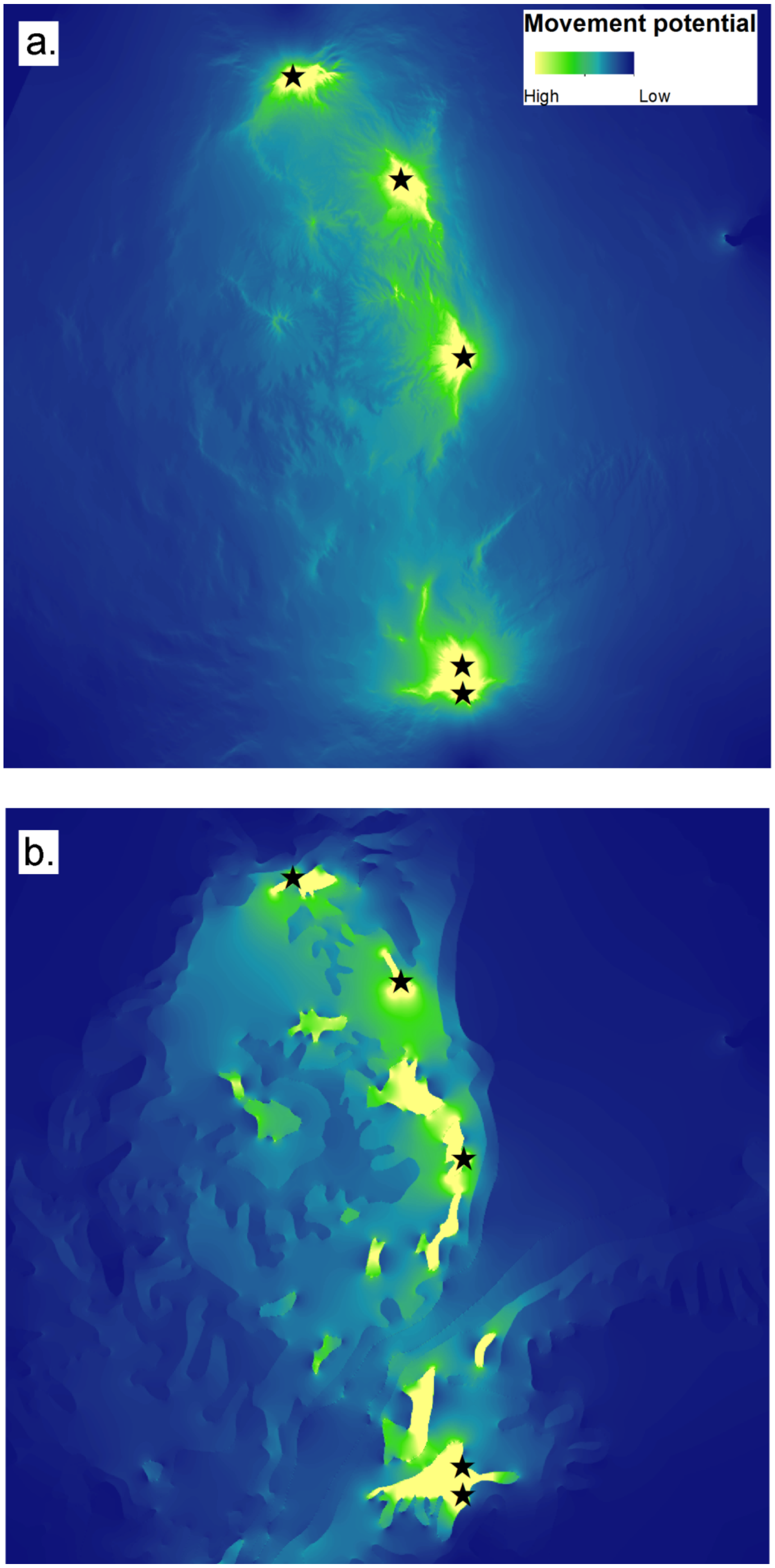

Figure S6 – Projected movement density based on the effect of altitude (a) and ecoregions (b) on genetic connectivity between *Plecotus balensis* population.

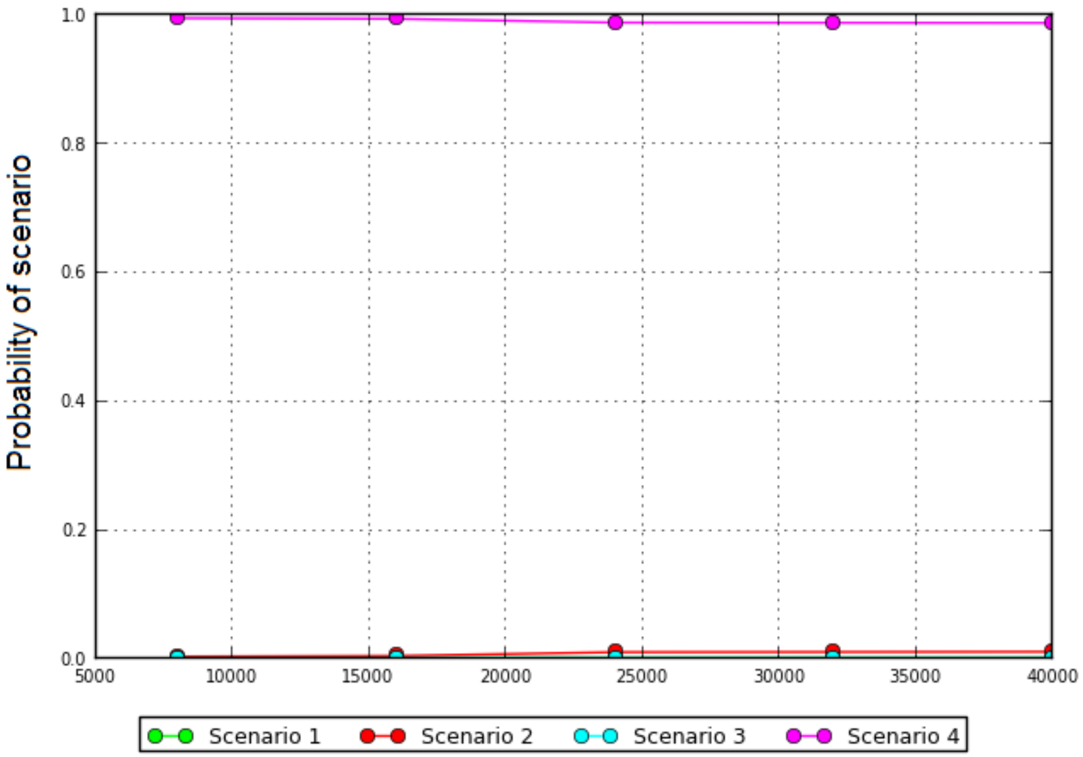

Figure S7 – Probability of scenarios compared in the approximate Bayesian computation model-based inference of *Plecotus balensis* demographic history. See Fig. B1 for scenario details.

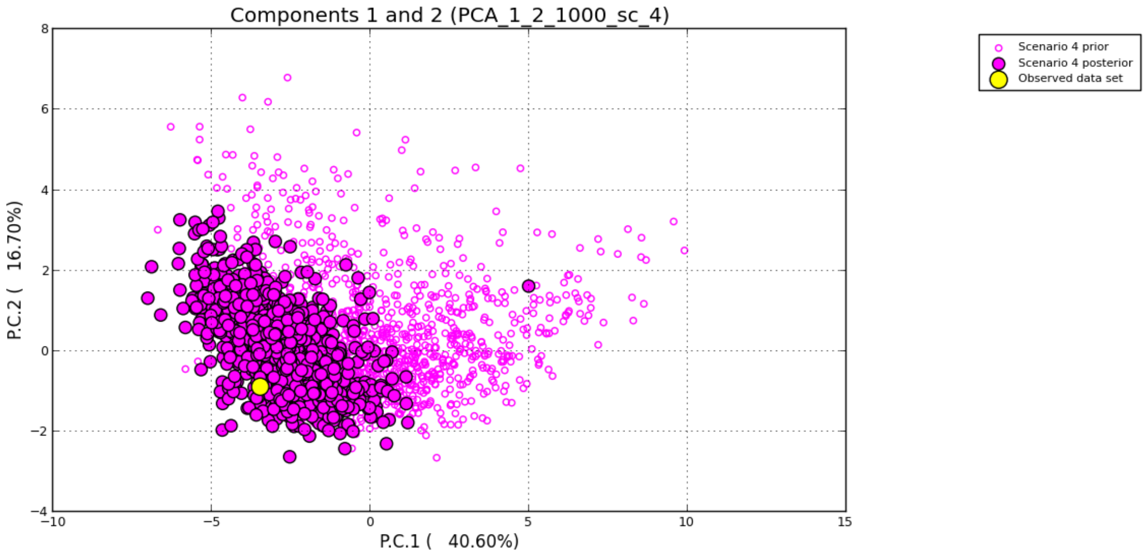

Figure S8 –DIYABC model checking analysis results for the most probable scenario (scenario 4). PCA plot showing where the observed dataset (yellow dot) falls relative to the simulated dataset generated from posterior parameter distributions (pink dots) and the original scenario 4 prior simulations (open pink circles). The observed dataset falls within the cloud of simulated points, indicating a good fit between observed and simulated datasets under the chosen priors.
